# Targeted plasma metabolomics reveals potential biomarkers of the elderly with mild cognitive impairment in Qingdao rural area

**DOI:** 10.3389/fnagi.2024.1511437

**Published:** 2024-12-18

**Authors:** Yuchi Meng, Murong Cheng, Hongyan Qu, Zhenxue Song, Ling Zhang, Yuanjun Zeng, Dongfeng Zhang, Suyun Li

**Affiliations:** Department of Epidemiology and Health Statistics, School of Public Health, Qingdao University, Qingdao, Shandong, China

**Keywords:** cognitive function, the elderly, metabolomics, mild cognitive impairment, MCI

## Abstract

**Introduction:**

Previous research has suggested a link between the onset of Alzheimer’s disease (AD) and metabolic disorder; however, the findings have been inconsistent. To date, the majority of metabolomics studies have focused on AD, resulting in a relative paucity of research on early-stage conditions such as mild cognitive impairment (MCI) underexplored. In this study, we employed a comprehensive platform for the early screening of individuals with MCI using high-throughput targeted metabolomics.

**Method:**

We included 171 participants including 124 individuals with MCI and 47 healthy subjects. Univariate statistical analysis was conducted using *t*-tests or Wilcoxon rank-sum tests, with *p*-values corrected by the Benjamini-Hochberg method. The screening criteria were set at FDR < 0.05 and fold change (FC) > 1.5 or < 0.67. Multivariate analysis was performed using orthogonal partial least squares discriminant analysis (OPLS-DA), where differential metabolites were identified based on variable influence on projection (VIP) scores (VIP > 1 and FDR < 0.05). Random forest analysis was used to further evaluate the ability of the metabolic data to distinguish effectively between the two groups.

**Results:**

A total of 14 differential metabolites were identified, leading to the discovery of a biomarker panel consisting of three plasma metabolites including uric acid, pyruvic acid and isolithocholic acid that effectively distinguished MCI patients from healthy subjects.

**Discussion:**

These findings have provided a comprehensive metabolic profile, offering valuable insights into the early prediction and understanding of the pathogenic processes underlying MCI. This study holds the potential for advancing early detection and intervention strategies for MCI.

## 1 Introduction

As the global population ages and the pace of aging accelerates, health issues among the elderly are drawing increasing attention. Age-related memory impairment (AAMI) is prevalent among older adults and frequently advances to mild cognitive impairment (MCI) or Alzheimer’s disease (AD). According to a recent national cross-sectional study of China, there are 15.07 million dementia patients aged 60 years and older, including 9.83 million with AD, 3.92 million with vascular dementia, and 1.32 million with other forms of dementia (Ren et al., 2022). In addition, the prevalence of MCI in people over 60 years of age was 15.5%, translating to 38.77 million people suffering from MCI (Jia et al., 2020). Currently, there is no effective treatment for AD, and cognitive decline remains an irreversible process. Therefore, early detection of biomarkers and timely intervention are crucial in delaying or preventing the progression of the disease to MCI or AD, ultimately enhancing the quality of life for the elderly.

Metabolomics, as a key branch of systems biology, has significantly contributions to the identification of biomarkers and the early prediction of diseases (Johnson et al., 2016; Patti et al., 2012). This field primarily focuses on the investigation of endogenous small-molecule metabolites (with a molecular weight less than 1,000) through both qualitative and quantitative approaches, shedding light on the dynamic changes in the body’s metabolite profiles in response to endogenous and exogenous factors and seek to identify metabolic pathways involved in disease progression. In recent years, the metabolomics studies on AD have provided valuable insights into its pathogenesis, and have led to the discovery of potential biomarkers for early diagnosis (Johnson et al., 2016; Xu et al., 2012; Wang et al., 2023a; Wang et al., 2023b). Many studies have reported metabolic profile differences between AD patients and healthy subjects (Graham et al., 2013; Liu et al., 2014). For instance, Graham et al. (2013) analyzed over 800 metabolite changes in postmortem brain tissue from 15 AD patients and 15 healthy controls, and identified five potential biomarkers for AD. Similarly, Li et al. (2023) performed metabolomics analysis and found significant metabolic differences in nine biomarkers, including lipids, amino acids, and sphingosine, in plasma samples from individuals with AD, MCI, and healthy subjects (Liu et al., 2014). Furthermore, (Wang et al., 2023b) conducted a metabolomic analysis using LC-MS/MS on 57 AD patients, 43 patients with MCI, and 62 cognitively healthy subjects. Their study identified a diagnostic panel for AD consisting of 30 metabolites, age, and APOE genotype, while the diagnostic panel for MCI included 45 metabolites, age, and APOE (Wang et al., 2023a). Similarly, Huo et al. (2020) analyzed 597 plasma samples and 111 postmortem brain samples, revealing that differential metabolites could effectively distinguish AD patients from individuals with normal cognitive function with high accuracy. While these findings support a strong link between metabolic alterations and AD, the results have shown inconsistency. In addition, the outcomes variables in the majority of previous metabolomics studies have concentrated on AD (Graham et al., 2013; Liu et al., 2014; Huo et al., 2020, Sato et al., 2012).

To date, few studies have specifically targeted MCI patients or developed a predictive model for early screening. Therefore, our purpose is to conduct plasma metabolomics studies in older adults using high-throughput targeted metabolomics with the aim of identifying different metabolic profiles and specific plasma biomarkers for early prediction of MCI.

## 2 Materials and methods

### 2.1 Study subjects

The study participants were recruited from 17 villages in the Jimo District of Qingdao City. Based on inclusion and exclusion criteria, 650 individuals participated in the survey, with only 3 failing to complete it, and the response rate was 99.54% (mean age: 73 years, female: 53.7%). Each respondent underwent a face-to-face interview using a questionnaire to gather demographic data, cognitive function information, and other environmental factors. Additionally, blood samples were collected from each participant simultaneously. The sample used in this study was the same as in our previously published research. The study was conducted by the Declaration of Helsinki and approved by the Ethical Committee of Qingdao University Medical College (ID: QDU-HEC-2023184). All patients in this study gave informed and written consent.

### 2.2 Cognitive function test

Cognitive function was evaluated using the Beijing version of the Montreal Cognitive Assessment (MoCA) (Nasreddine et al., 2005). MoCA is an assessment tool developed by Nasreddine in 2004 for rapid screening for MCI. The cognitive areas assessed by the MoCA scale include seven parts: visuospatial ability with a score of 0–5, naming ability with a score of 0–3, attention ability with a score of 0–6, language ability with a score of 0–3, abstract ability with a score of 0–2, delayed recall ability with a score of 0–5 and orientation ability with a score of 0–6. The total score of the scale is 30 points, and the test results show that the normal value is ≥ 26 points and < 26 points are defined as the optimum cutoff point for a definition of MCI (Kang et al., 2018). If the number of years of education is less than 12, 1 point can be added. Individuals with scores ≥ 26 were categorized as the control group. The scale provides a comprehensive assessment of respondents’ cognitive function across various dimensions, with higher scores indicating superior cognitive function.

Our study comprised a total of 647 participants, including 411 individuals were diagnosed with MCI and 236 healthy controls. Blood samples were collected from each participant. However, due to quality control issues, such as hemolysis, only 271 plasma samples met the quality criteria. From this subset, 171 samples were randomly selected for metabolomic analysis, comprising 47 healthy individuals and 124 patients. The Cronbach’s α coefficient of MoCA Beijing is 0.74 in the survey population.

### 2.3 High throughput targeted metabolomics procedures

The H650 Medical high-throughput targeted metabolomics approach was adopted in this study (Lv et al., 2023; Luo et al., 2023; Qin et al., 2022). In comparison with commonly targeted metabolomics, this method employs complete standards and enables the detection of a wide variety and a large number of metabolites. Absolute qualitative and quantitative analyses of approximately 650 functional metabolites in medical research can be conducted. The high-throughput targeted metabolomics approach can simultaneously detect multiple sample types and substances, and obtain accurate qualitative and quantitative results without the need for re-verification. The H650 medical high-throughput targeted metabolomics approach, utilizing full standards for method development, enables absolute qualitative and quantitative analysis of approximately 650 types of functional metabolites in medical research. In this study, the quantitative gold standard multiple reaction monitoring (MRM) method was employed. MRM is a mass spectrum signal acquisition technique based on known or assumed information. MRM can be quantified through the internal standard method. The internal standard method is extensively applied in the quantitative analysis of UPLC-MS/MS. The metabolites encompass amino acids, organic acids, carbohydrates, fatty acids, lipids, nucleotides, vitamins, coenzymes, neurotransmitters, bile acids, polyamines, and related metabolites. It can precisely qualitative and quantitative, and extensively cover the relevant pathways of medical research. Specific analysis methods for the H650 Medical high-throughput targeted metabolomics approach can be found on this website.^1^

#### 2.3.1 Sample collection and extraction

During the questionnaire period, fresh blood samples were collected from all participants. Blood samples were obtained following a 12-h fasting period. The blood samples were immediately separated at 3,000 rpm for 10 min, and plasma samples (1 mL each) were obtained. The plasma samples were rapidly frozen with dry ice and stored at −80°C within 4 h post-collection. Ultra-high-performance liquid chromatography-mass spectrometry (LC/MS) was employed for further analysis.

Before the experiment, all plasma samples were thawed at 4°C. The pre-cooled methanol, acetonitrile, and water solution were then added in a 2:2:1 ratio and vortexed uniformly. Subsequently, the samples were stored at −20°C for 10 min, followed by centrifugation at 14,000 × *g* for 20 min at 4°C. The supernatant was collected from the sample and dried under a vacuum. A 100 μL aqueous acetonitrile was added and vortexed homogeneously for mass spectrometry analysis. Finally, the samples were centrifuged at 14,000 × *g* for 15 min at 4°C and the supernatant was injected for analysis.

#### 2.3.2 UHPLC–MS/MS analysis

The analytes were separated on HILIC (Waters UPLC BEH Amide column, 2.1 mm, 100 mm, 1.7 μm) and C18 columns (Waters UPLC BEH C18-2.1 100 mm, 1.7 μm). The C18 column serves as a nonpolar stationary phase that can retain nonpolar compounds in reversed-phase chromatography, for example, lipids and lipid-like molecules, phenylpropanoids and polyketides. The HILIC column is a polar stationary phase that can retain polar compounds in hydrophilic interaction chromatography, for example, sugars, amino acids, organic acids and nucleotides.

The column temperature was 35°C, the flow rate was 0.3 mL/min, and the sample size was 2 μL. The mobile phases comprised of solvent A (2 mM ammonium formate + 10% acetonitrile + 90% water + 40% isopropanol) and solvent B (acetonitrile +0.4% formic acid). The gradient elution conditions were described as follows: 0–1 min, 85% solvent B; 1–3 min, 85–80% solvent B; 3–4 min, 80% solvent B; 4–6 min, 80–70% solvent B; 6–10 min, 70–50% solvent B; 10–15.5 min, 50% solvent B; 15.5–15.6 min, 50–85% solvent B, 15.6–23 min, 85% solvent B. The C18 column temperature was 40°C, the flow rate was 0.4 mL/min, and the sample size was 2 μL. The mobile phases comprised of solvent A (water +5 mM ammonium acetate) and solvent B (99.5% acetonitrile). The gradient elution conditions were described as follows: 0–5 min, 5–60% solvent B; 5–11 min, 60–100% solvent B; 11–13 min, 100% solvent B; 13–13.1 min, 100–5% solvent B; 13.1–16 min, 5% solvent B. To minimize the impact of fluctuations in instrument detection signals, the samples are analyzed continuously and in a random order. Quality control (QC) samples were incorporated into the sample queue to assess the stability of the system and ensure the reliability of experimental data. In this study, a high-throughput metabolome-targeting approach was utilized to qualitatively and quantitatively analyze over 600 common metabolites relevant to medical research including metabolites such as carbohydrates, organic acids, amino acids, bile acids, indoles, purine nucleotides, lipids, and other compounds. The aim was to facilitate the understanding of disease development in the medical field, identify potential disease biomarkers, and predict disease prognosis. Analyses were performed using a UHPLC (1,290 Infinity LC, Agilent Technologies) coupled to a QTRAP MS (AB 6500+, AB Sciex) at Shanghai Applied Protein Technology Co., Ltd.

### 2.4 Covariates

Covariates include age, gender (male/female), education (≤ 12 years/> 12 years), occupation status (farmers/others), marital status (married/unmarried or widowed), hypertension (yes/no), diabetes (yes/no), stroke (yes/no), coronary heart disease (yes/no), body mass index (BMI). Participants with prevalent hypertension, diabetes, stroke and coronary heart disease were identified if they had a previous self-reported physician diagnosis and/or treatment for the respective condition.

### 2.5 Data processing and statistical analysis

The MultiQuant or Analyst software was utilized for extracting peaks from the raw data of targeted MRM, calculating the ratio of each substance’s peak area to that of the internal standard. Concentrations were then determined based on standard curves. Metabolites with more than 80% missing values were removed, the remaining missing values were imputed using the k-nearest neighbor (KNN) method. Subsequently, the data were then normalized by the sum method and analyzed using R (4.3.1), Stata (version 15.0), and MetaboAnalyst 6.0.^2^

For data that followed a normal distribution, continuous variables were presented as mean ± standard deviation, and those that did not follow a normal distribution were expressed as median (interquartile range). While categorical data were displayed as counts and percentages. Comparisons of continuous variables between the two groups were performed using independent *t*-tests or Wilcoxon rank-sum tests, depending on the data distribution. Categorical variables were compared using Chi-square or Fisher’s exact tests. To account for multiple comparisons, the Benjamini-Hochberg false discovery rate (FDR) method was applied, with an FDR *q*-value threshold set at 0.05 (Hochberg and Benjamini, 1990). Additionally, the fold change was calculated based on the mean metabolite levels in the MCI group relative to the control group.

The principal component analysis (PCA), partial least squares discriminant analysis (PLS-DA) and orthogonal partial least squares discriminant analysis (OPLS-DA) models were internally validated using 7-fold cross-validation. To prevent overfitting, model validation was conducted using 200 random permutations of the sample group labels. The explanatory and predictive power of the models was assessed using R2Y and Q2Y, respectively. Metabolites were considered differentially abundant if they met the following criteria: VIP (variable importance in the projection) score > 1 with FDR < 0.05.

Metabolic pathway analysis was conducted using MetaboAnalyst 6.0. The Pearson correlation coefficient was calculated to assess the relationships between the differential metabolites, and a correlation heatmap was generated for visual representation of these associations. Hierarchical cluster analysis (HCA) and clustering heat map were performed on the differential metabolites using R (“pheatmap” package). Logistic regression and the receiver operating characteristic (ROC) analysis were used to identify a biomarker panel for diagnosing MCI, adjusting for other covariates such as age, gender, education and occupation. The area under the curve (AUC) was used to assess the predictive performance of the identified metabolic biomarkers for MCI. Additionally, the Delong test was applied to compare the predictive values of different prediction models.

## 3 Results

### 3.1 Participant characteristics

Table 1 presents the basic characteristics of 171 participants in targeted metabolomics, and the sample was categorized by their baseline cognitive status. In comparison to the control group, individuals with MCI were found to be significantly older, have lower levels of education, and the proportion of farmers is higher. In addition, participants in the MCI group presented lower cognitive scores (*p* < 0.05). Compared to the control group, individuals with cognitive impairment have a higher proportion of being married. No statistically significant differences were observed between the two groups in terms of the distribution of gender, marital status, hypertension, diabetes, stroke, coronary heart disease, or mean BMI.

**TABLE 1:** Baseline characteristics of two groups of people by cognitive function.

Characteristics	MCI group (*N* = 124)	Control group (*N* = 47)	*P*-value
Age (years)^a^	74.58±5.79	70.53±5.32	0.000
Gender [*n* (%)]^b^			0.210
Male	45 (36.3%)	22 (46.8%)	
Female	79 (63.7%)	25 (53.2%)	
Education [*n* (%)]^b^			0.002
≤ 12 years	122 (98.3%)	41 (87.2%)	
> 12 years	2 (1.7%)	6 (12.8%)	
Occupation status [*n* (%)]^b^			0.028
Farmers	101 (83.5%)	32 (68.1%)	
Others	20 (16.5%)	15 (31.9%)	
Marital status [*n* (%)]^b^			0.349
Married	91 (74.0%)	38 (80.9%)	
Unmarried or widowed	32 (26.0%)	9 (19.1%)	
Hypertension [*n* (%)]^b^			0.493
Yes	52 (41.9%)	17 (36.2%)	
No	72 (58.1%)	30 (63.8%)	
Diabetes [*n* (%)]^b^			0.425
Yes	16 (12.9%)	4 (8.5%)	
No	108 (87.1%)	43 (91.5%)	
Stroke [*n* (%)]^b^			0.585
Yes	8 (6.5%)	2 (4.3%)	
No	116 (93.5%)	45 (95.7%)	
Coronary heart disease [*n* (%)]^b^			0.295
Yes	18 (14.5%)	4 (8.5%)	
No	106 (85.5%)	43 (91.5%)	
BMI^a^	25.93 ± 0.38	25.27 ± 0.64	0.670
Visuospatial ability^a^	1.91 ± 1.40	3.74 ± 1.11	0.000
Naming ability^a^	2.86 ± 0.48	3.00 ± 0.00	0.028
Attention ability^a^	3.94 ± 1.62	5.80 ± 0.50	0.000
Language ability^a^	2.52 ± 0.81	2.97 ± 0.15	0.000
Abstract ability^a^	0.81 ± 0.77	1.51 ± 0.66	0.000
Delayed recall ability^a^	2.00 ± 1.70	4.34 ± 0.84	0.000
Orientation ability^a^	4.84 ± 1.30	5.61 ± 0.64	0.000
MoCA total scores^a^	18.9 ± 4.30	27.02 ± 1.73	0.000

^a^Mean ± standard deviation was used for statistical description, and *t*-test was used to calculate *P*-value for inter-group comparison.

^b^*n* (%) was used for description, and Fisher’s precision test or Chi-square test were used to calculate *P*-values for inter-group comparison.

### 3.2 Metabolic profiles of MCI patients

To compare the metabolic profiles between individuals with MCI and those in the healthy subjects, we conducted targeted quantitative validation using UHPLC-qTRAP MS methods. Green dots indicate the center clustering of QC samples, indicating that the mass accuracy of the mass spectrometry data meets the requirements (Figure 2A).

**FIGURE 1:**
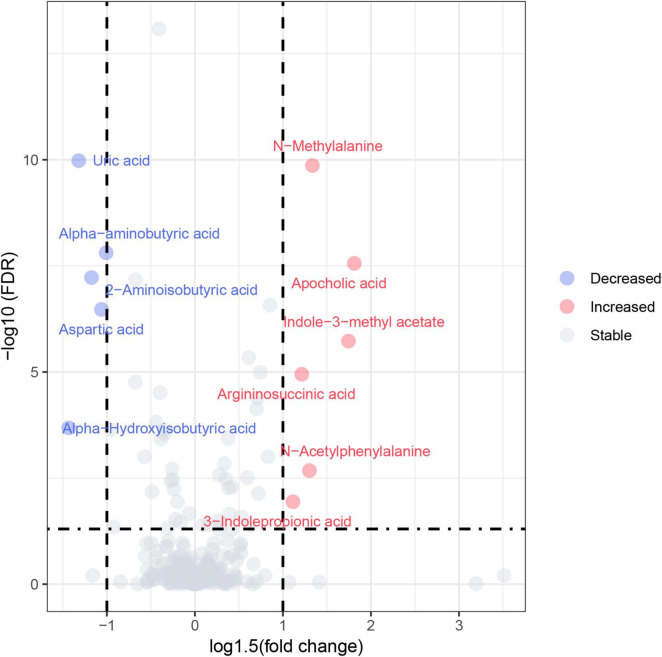
Volcano plot. Red circles represented increased metabolites and blue circles represented decreased metabolites.

404 metabolites were detected by the targeted metabolomics method. After removing metabolites with more than 80% missing data, the KNN method was used to interpolate the remaining metabolites, resulting in a total of 248 metabolites left (Shahjaman et al., 2021). The detected metabolites were categorized into 9 classes, with lipids and lipid-like molecules constituting the largest proportion. Based on the criteria of FC > 1.5 or FC < 0.67 and FDR value < 0.05, a total of 11 differential metabolites were identified in the univariate statistical analysis, compared with the control group, 6 metabolites were significantly increased and 5 metabolites were significantly decreased in MCI group (Table 2). The volcano plot was presented in Figure 1. In addition, we used logistic regression to adjust for gender as a covariate to analyze the relationship between univariate differential metabolites and cognition (Supplementary Table 1). After adjusting for gender, all variables, except for 3-Indolepropionic acid, remained significantly associated with cognition.

**TABLE 2:** Univariate statistical analysis screened for differential metabolites between participants with MCI with and control group.

Metabolites	m/z	RT (min)	MCI/CON		
			**FC^a^**	**VIP^b^**	**State^c^**
Alpha-hydroxyisobutyric acid	103.0/57.0	2.90	0.56	0.24	↓**
Uric acid	167.1/124.1	6.65	0.59	1.11	↓**
2-Aminoisobutyric acid	104.0/58.0	5.90	0.62	0.24	↓**
Aspartic acid	132.0/88.0	8.10	0.65	0.25	↓**
Alpha-aminobutyric acid	104.0/58.1	5.80	0.66	0.19	↓**
3-Indolepropionic acid	187.9/59.1	5.43	1.57	0.22	↑*
Argininosuccinic acid	291.1/70.2	9.13	1.64	1.11	↑**
N-Acetylphenylalanine	206.0/164.0	4.23	1.69	0.02	↑**
N-Methylalanine	104.0/58.0	5.38	1.72	0.38	↑**
Indole-3-methyl acetate	190.0/130.0	5.77	2.03	0.11	↑**
Apocholic acid	389.4/389.4	9.35	2.08	0.37	↑**

CON, control; FC, fold change; VIP, variable influence on projection; RT, retention time.

^a^FC value was calculated as the ratio of the average mass response (area) between the two groups (FC value = MCI/control).

^b^Only metabolites with FC > 1.5 or FC < 0.67 and FDR less than 0.05 were deemed statistically significant in univariate statistical analysis.

^c^↑ Represented a significant increase in metabolite levels in MCI group compared with the control group, ↓ represented a significant decrease in metabolite levels in MCI group compared with the control group;

*FDR < 0.05,

**FDR< 0.01.

**FIGURE 2:**
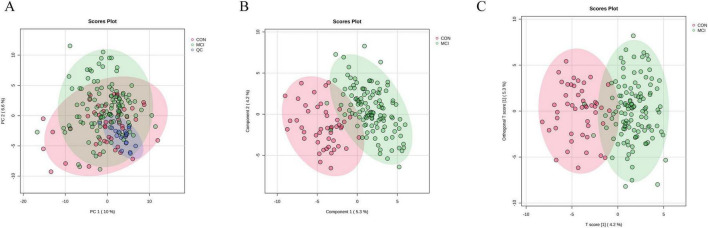
Results of multivariate statistical analysis for targeted metabolomics profiling. **(A)** Score plot of PCA. The respective model interpretability for the X variable datasets was R2X = 0.48, model predictability Q2 = 0.18. **(B)** Score plot of PLS-DA. The respective model interpretability for the X and Y variable datasets was R2X = 0.13 and R2Y = 0.73, model predictability Q2 = 0.55. **(C)** Score plot of OPLS-DA. The respective model interpretability for the X and Y variable datasets was R2X = 0.13 and R2Y = 0.73, model predictability Q2 = 0.59.

To further visualize and classify metabolic profiles between individuals with MCI and healthy controls, various multivariate analyses such as PCA, PLS-DA and OPLS-DA were employed to simplify the target metabolomics data and compare metabolic differences. The PCA showed no significant difference between the MCI group and the control group (Figure 2A). Our results from the PLS-DA and OPLS-DA revealed a proper separation of all patients with MCI from healthy controls (Figures 2B, C). These results demonstrate a reliable distinction of metabolic changes between participants with MCI and controls. As the permutation retention gradually declined, both R2 and Q2 of the random model declined, indicating that the original model did not overfit and possessed good robustness.

### 3.3 Differential metabolite identification and analysis

**FIGURE 3:**
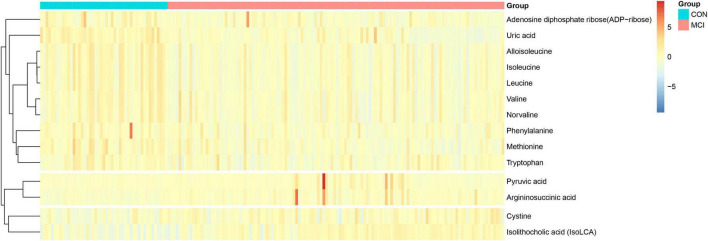
The hierarchical clustering heat map of the metabolites. The rows represent the 14 metabolites, and the columns represent samples in the control and participants with low cognitive function; Red and blue indicate increased and decreased, respectively.

For the multivariate statistical analysis, 14 metabolites were screened based on the criteria of VIP > 1 and FDR < 0.05 (Table 3). These metabolites were further categorized into 7 subclasses, with amino acids representing the largest proportion, including aspartic acid, leucine, valine, etc. Four metabolites were found to be significantly increased and 10 metabolites were significantly decreased in the MCI group. Except for cystine and argininosuccinic acid, the remaining amino acids were significantly decreased. The results showed that common differential metabolites meeting the univariate and multivariate criteria (FC > 1.5 or < 0.67, FDR < 0.05, VIP > 1) included arginine succinic acid and uric acid. Specifically, the hierarchical cluster heatmap indicated that five essential amino acids, namely valine, phenylalanine, leucine, isoleucine, and methionine, along with two free amino acids, alloisoleucine and norvaline, were significantly increased in the MCI group in Figure 3.

**TABLE 3:** Key differential expressed metabolites between participants with MCI with and control group.

Metabolites	m/z	RT (min)	MCI/CON		
			**FC^a^**	**VIP^b^**	**State^c^**
Pyruvic acid	87.1/43.1	1.02	1.24	6.93	↑**
Cystine	241.1/151.9	8.45	1.15	4.16	↑**
Tryptophan	205.2/188.1	3.53	0.92	2.59	↓**
ADP-ribose	560.0/348.0	8.38	0.69	2.52	↓**
Leucine	132.1/86.2	3.68	0.87	2.42	↓**
IsoLCA	375.5/375.5	9.93	1.41	2.35	↑**
Valine	118.1/55.1	4.79	0.90	2.34	↓**
Phenylalanine	166.1/120.1	3.47	0.90	2.30	↓**
Isoleucine	132.1/86.2	4.01	0.87	2.28	↓**
Alloisoleucine	132.1/86.1	4.00	0.85	1.69	↓**
Methionine	150.1/133.0	4.19	0.86	1.56	↓**
Norvaline	118.1/72.2	4.51	0.91	1.19	↓**
Argininosuccinic acid	291.1/70.2	9.13	1.64	1.11	↑**
Uric acid	167.1/124.1	6.65	0.59	1.11	↓**

CON, control; FC, fold change; VIP, variable influence on projection; RT, retention time.

^a^FC value was calculated as the ratio of the average mass response (area) between the two groups (FC value = MCI/control).

^b^Only metabolites with VIP values greater than 1.0 and FDR less than 0.05 were deemed statistically significant in multivariate statistical analysis.

^c^↑ Represented a significant increase in metabolite levels in MCI group compared with the control group, ↓ represented a significant decrease in metabolite levels in MCI group compared with the control group;

**FDR < 0.01.

### 3.4 Correlation and functional pathway analysis of differentially abundant metabolites

Correlation analysis can assist in measuring the metabolic proximities of significantly different metabolites, thereby enhancing our understanding of the mutual regulatory relationships between metabolites during biological state changes. This facilitates further insight into the intricate dynamics of metabolic processes. We conducted correlation analysis for 14 differential metabolites, and the results of the correlation analysis were shown in Supplementary Figure 1, with blue representing positive correlation and red representing negative correlation.

Notably, isoleucine and alloisoleucine which both belong to the branched-chain amino acid (BCAAs) were positively correlated with valine (correlation coefficient = 0.87, *P* < 0.001; correlation coefficient = 0.88, *P* < 0.001). We observed a significant positive correlation between changes in the levels of valine and norvaline (correlation coefficient = 0.98; *P* < 0.001). leucine and alloisoleucine were positively correlated with isoleucine (correlation coefficient = 0.99, *P* < 0.001; correlation coefficient = 0.99, *P* < 0.001).

**FIGURE 4:**
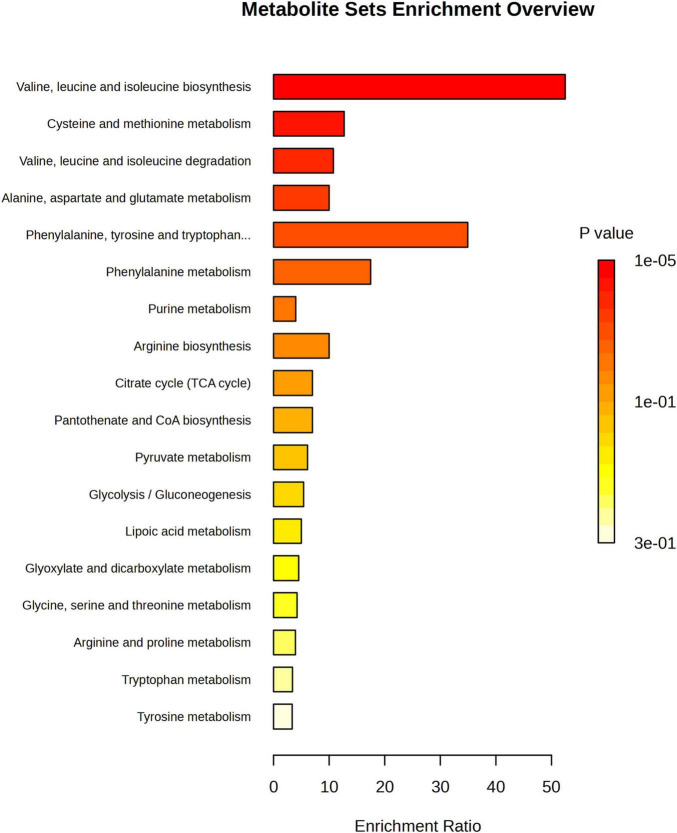
Metabolic pathway enrichment analysis bar chart; The enrichment ratio is the ratio of metabolites enriched in the pathway to all metabolites in the pathway.

**FIGURE 5:**
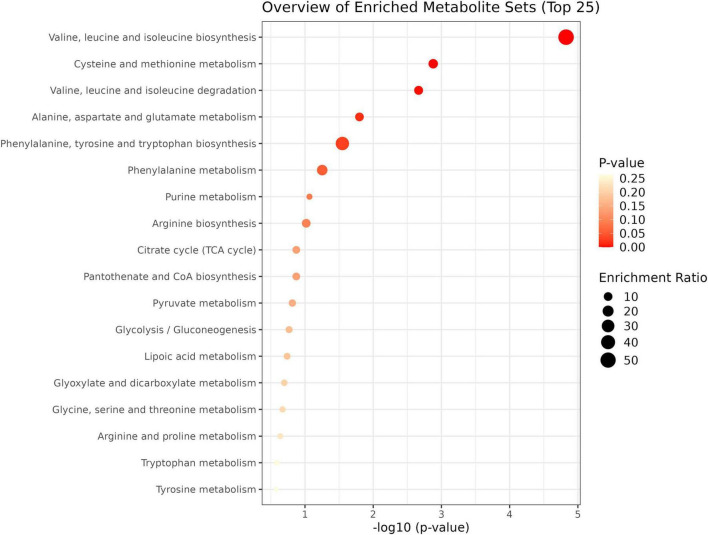
Metabolic pathway enrichment analysis bubble diagram. The node color is based on its *p*-value, and the node radius is based on their pathway impact values.

To investigate the differential metabolite pathways associated with MCI, pathway enrichment analyses were performed. The bubble diagram and bar diagram of metabolic pathways were depicted in Figures 4, 5. It was evident that 14 metabolites were enriched across 18 metabolic pathways, including the biosynthesis of valine, leucine and isoleucine biosynthesis; the metabolism of cysteine and methionine; the degradation of valine, leucine and isoleucine; the metabolism of alanine, aspartate and glutamate and the biosynthesis of phenylalanine, tyrosine and tryptophan (*p* < 0.05). Among the 14 metabolites, there were nine amino acids, which were implicated in eight metabolic pathways. They are significantly involved in the biosynthesis of valine, leucine, and isoleucine; the degradation of valine, leucine, and isoleucine; the metabolism of cysteine and methionine and the metabolism of phenylalanine (*p* < 0.05).

### 3.5 Potential biomarker panel for predicting of MCI

The 14 differential metabolites were further screened to identify key metabolites that could predict cognitive decline for model development. In order to explore a biomarker panel for diagnosis of MCI, logistic regression and ROC analysis were used to disclose the most qualified metabolic candidate. As a result, three key metabolites (uric acid, pyruvic acid and isolithocholic acid) were identified as biomarkers. Box plots for the three biomarkers showed that uric acid levels were significantly lower, while pyruvic acid and isolithocholic acid levels were significantly higher in individuals with MCI compared to the control group.

**FIGURE 6:**
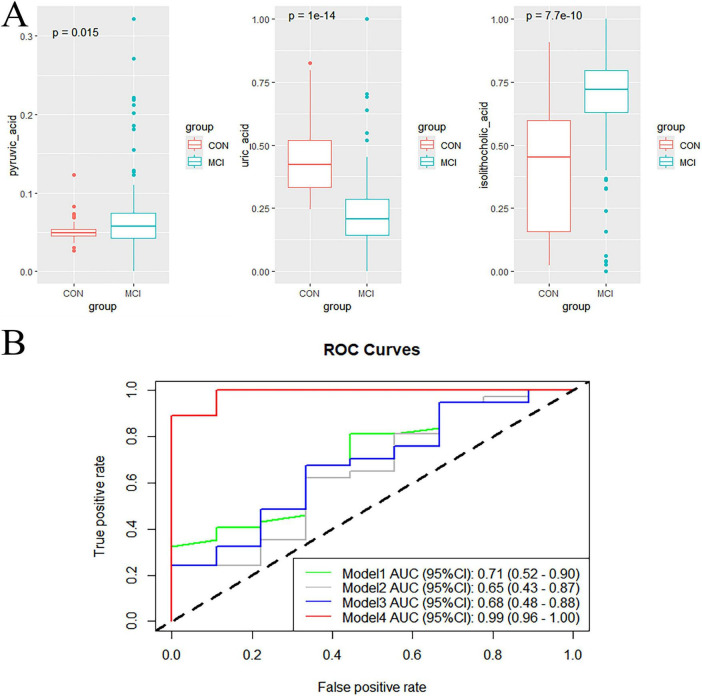
**(A)** Box plots of three normalized potential biomarkers. Samples were compared using the Wilcoxon rank sum test. **(B)** ROC curves of the predictive models. The logistic regression models were adjusted for various covariates as follows: Model 1 included adjustments for age, gender, education, and occupation; Model 2 incorporated additional adjustments for marital status and body mass index (BMI); Model 3 further included hypertension, diabetes, stroke, and coronary heart disease; and Model 4 was adjusted for age, gender, education, occupation, as well as biochemical markers such as uric acid, pyruvic acid, and isolithocholic acid.

Three logistic regression models were constructed to assess the best combination of demographic factors for predicting MCI. Compared to Model 2 and Model 3, Model 1, including age, sex, education, and occupation, exhibited the best predictive ability for MCI, with an AUC of 0.71 (95% CI: 0.52–0.91). When the three metabolites were added to Model 1, the performance significantly improved, yielding an AUC of 0.99 (95% CI: 0.96–1.00) as shown in Figure 6. The results of the DeLong test indicated a significant difference in the AUC between the two models (*p* < 0.01). These findings demonstrated that the combination of uric acid, pyruvic acid, and isolithocholic acid had a strong predictive potential for cognitive decline in MCI progression. Graphical abstract was shown in the Supplementary Figure 2.

## 4 Discussion

In this study, targeted metabolomics was conducted to analyze the changes in plasma metabolite levels in 124 participants with MCI and 47 controls. The results demonstrated that the metabolic levels in participants with MCI differ from those of controls. Specifically, univariate and multivariate statistical analyses revealed 11 and 14 different metabolites, respectively. Further pathway analysis indicated that these altered metabolites were primarily involved in several key metabolic pathways, including the biosynthesis of valine, leucine and isoleucine biosynthesis; the metabolism of cysteine and methionine; the degradation of valine, leucine and isoleucine; the metabolism of alanine, aspartate and glutamate and the biosynthesis of phenylalanine, tyrosine and tryptophan (*p* < 0.05).

Additionally, we found that a subset of metabolites with differential abundance could potentially serve as biomarkers for MCI. The integration of three metabolites including uric acid, pyruvic acid and isolithocholic acid significantly improved the performance of predicting MCI progression compared to models that relied solely on demographic characteristics. In the absence of gold-standard diagnostic procedures, diagnostic accuracy can be greatly improved by utilizing a collaborative panel of diagnostic markers with multiple components, based on clinical manifestations and auxiliary examinations (Yang et al., 2013).

In this study, we observed significant differences in the metabolic profile of peripheral blood between subjects with MCI and healthy controls. Specifically, our findings revealed notable changes in amino acid levels, with six essential amino acids (leucine, tryptophan, methionine, isoleucine, phenylalanine and valine) showing a significant decline in the MCI group. These results were consistent with previous research indicating that cognitive decline was associated with decreased levels of nine essential amino acids (Ikeuchi et al., 2022; Aquilani et al., 2023). Furthermore, three BCAAs -leucine, isoleucine and valine-were also found to be significantly decreased in the MCI group consistent with earlier findings (Ikeuchi et al., 2022). A metabolomics study based on eight prospective cohorts revealed that lower serum concentrations of BCAAs were linked to an increased risk of all types of dementia (Tynkkynen et al., 2018). Similarly, a study utilizing circulating plasma metabolomics data from the UK Biobank reported lower plasma levels of BCAAs associated with the development of dementia (Zhang et al., 2022).

This association may be due to the potential of BCAA concentrations in the blood to reflect AD pathology, such as brain inflammation or oxidative stress (Ikeuchi et al., 2022). Previous research has shown that the protein among individuals with dementia was significantly lower than that of healthy elderly individuals (Nes et al., 1988; Sanders et al., 2018; Thomas et al., 1986). Moreover, higher protein intake in older adults has been linked to a reduced risk of MCI (Goodwin et al., 1983; La Rue et al., 1997), and to decrease accumulation of Aβ accumulation in the brain (Fernando et al., 2018). In the MCI group, plasma levels of other free amino acids were also significantly reduced, including alpha-aminobutyric acid, aspartic acid and 2-aminoisobutyric acid. Research has shown that alpha-aminobutyric acid, a non-protein amino acid derived from methionine, threonine, serine, and glycine, may play a role in regulating macrophage polarization and function through metabolic and epigenetic pathways, suggesting potential for treating inflammatory diseases (Li et al., 2023). Tryptophan, an essential amino acid necessary for protein synthesis in humans, is also the sole precursor of the neurotransmitter serotonin, which regulates various physiological processes related to emotion, memory, and learning. Furthermore, evidence from major drug treatments that delay the progression of AD suggested a legitimate role of gut microbes and tryptophan metabolites in the development of this condition (Roth et al., 2021).

A study on the gut microbiome has revealed an association between bile acid metabolism and cognitive dysfunction (MahmoudianDehkordi et al., 2019). Consistent with our plasma-targeted metabolomics findings, we observed significantly elevated levels of apocholic acid and isolithocholic acid in the MCI group. Research has shown that bile acid cytotoxicity is linked to membrane damage, oxidative stress, and apoptosis (Amaral et al., 2009). Another study also suggested that bile acid metabolism disorders were associated with AD, as they can contribute to cholestatic liver disease, dyslipidemia, fatty liver disease, cardiovascular disease, and diabetes, conditions that were directly or indirectly associated with the risk of cognitive decline (Kim et al., 2016; Sims et al., 2017; Hall et al., 2017).

Abnormal pyruvate metabolism has been implicated in several conditions, including cancer, heart failure, and neurodegeneration (Gray et al., 2014). Pyruvic acid, the end product of anaerobic glycolysis, was found to be significantly upregulated in the MCI group, indicating that cognitive decline may be associated with an anoxic state. Uric acid has long been associated with a negative reputation (Tana et al., 2018; Huang et al., 2022). However, recent studies have demonstrated that uric acid may play an active role in neurotoxicity or neuroprotection. These effects were thought to be linked to oxidative stress or inflammatory processes within the central nervous system, as well as other somatic or systemic diseases (Mijailovic et al., 2022). In line with this, our findings showed that uric acid levels were lower in the MCI group compared to the control group. This difference may reflect the antioxidant properties of uric acid, which could potentially play a neuroprotective role in AD (Tana et al., 2018; Mijailovic et al., 2022).

There were some advantages in this research. First, it focused on the early screening of individuals with MCI, allowing for the potential identification of biomarkers for early detection. Second, the sample size was sufficiently large and the use of plasma samples for analysis was less invasive and more cost-effective for patients. Additionally, targeted metabolomics offered several advantages, including high specificity, excellent sensitivity, and precise quantification.

However, there were also some limitations in this study. Firstly, the study utilized cross-sectional data, which limited the assessment of the temporal stability of biomarkers in MCI; future research will incorporate longitudinal data to examine the temporal dynamics of these metabolites. Secondly, other factors that may influence MCI, such as diet information and genetic variables, and clinical biomarker NfL were not investigated in this study. Furthermore, the present study comprised only 47 controls compared to 124 MCI patients, the imbalance sample size between groups might introduce bias, limiting the representativeness and reliability of control comparisons. Lastly, the majority of differential metabolites identified in this research lacked specificity, and our comprehension of these altered metabolites and their underlying mechanisms was still in its early stages.

## 5 Conclusion

In conclusion, we have identified significant changes in the metabolic profiles of both the MCI and control groups. These alterations involve key metabolic pathways, including the biosynthesis of valine, leucine and isoleucine biosynthesis; the metabolism of cysteine and methionine; the degradation of valine, leucine and isoleucine; the metabolism of alanine, aspartate and glutamate and the biosynthesis of phenylalanine, tyrosine and tryptophan (*p* < 0.05). Our findings confirmed that the prediction of MCI involves multiple biomarkers rather than a single biomarker. Specifically, we identified three metabolites, including uric acid, pyruvic acid and isolithocholic acid that may play a key role in the early prediction of MCI.

## Data Availability

The raw data supporting the conclusions of this article will be made available by the authors, without undue reservation.

## Footnotes

1
https://www.aptbiotech.com/asg/1062

2
https://www.metaboanalyst.ca/
