# Supplementary material for: Targeted plasma metabolomics reveals potential biomarkers of the elderly with mild cognitive impairment in Qingdao rural area
Source: Front Aging Neurosci. 2024 Dec 18;16:1511437. doi: 10.3389/fnagi.2024.1511437 (PMC11688483; doi:10.3389/fnagi.2024.1511437)
Supplement: Supplementary file 1 [file Table_1.docx]

***Supplementary Material***

Table S1. Association between univariate differential metabolites and cognition was analyzed using logistic regression^a^

| Metabolite name | OR (95%CI) | P |
| --- | --- | --- |
| Alpha-Hydroxyisobutyric acid | 0.92 (0.88-0.95)b | <0.001 |
| Uric acid | 0.99 (0.98-0.99)b | <0.001 |
| 2-Aminoisobutyric acid | 0.85 (0.80-0.91)b | <0.001 |
| Aspartic acid | 0.87 (0.83-0.92)b | <0.001 |
| Alpha-aminobutyric acid | 0.77 (0.70-0.85)b | <0.001 |
| 3-Indolepropionic acid | 1.00 (0.99-1.00)b | 0.560 |
| Argininosuccinic acid | 1.01 (1.01-1.02)b | <0.001 |
| N-Acetylphenylalanine | 1.29 (1.16-1.67)c | <0.001 |
| N-Methylalanine | 1.07 (1.04-1.10)b | <0.001 |
| Indole-3-methyl acetate | 1.36 (1.17-1.59)b | <0.001 |
| Apocholic acid | 1.11 (1.06-1.15)b | <0.001 |

^a^ Adjust the gender variable

^b^ The OR value for every 10-unit change in the metabolite

^c^The OR value for every 1-unit change in the metabolite


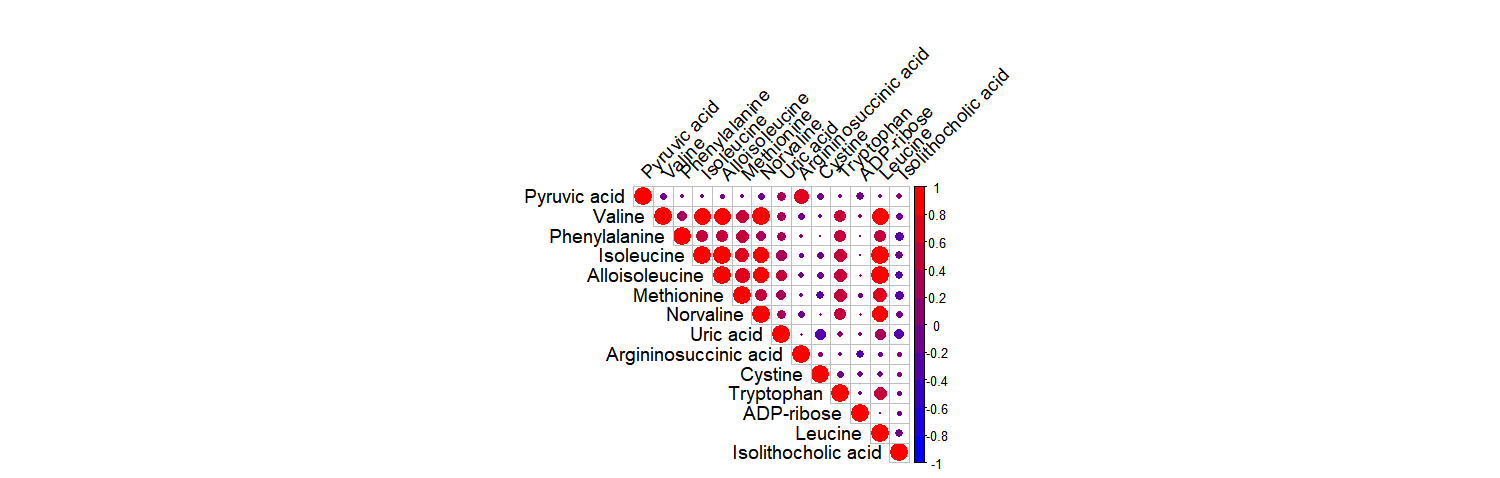


**FIGURE S1** Correlation heat map of 14 differential metabolites. Correlation analysis was conducted by calculating the Pearson’s correlation coefficient of two metabolites; The vertical bar on the right represents the value of the correlation coefficient, closer to red is a positive correlation, while closer to blue is a negative correlation, the size of the circle represents the size of the correlation coefficient.


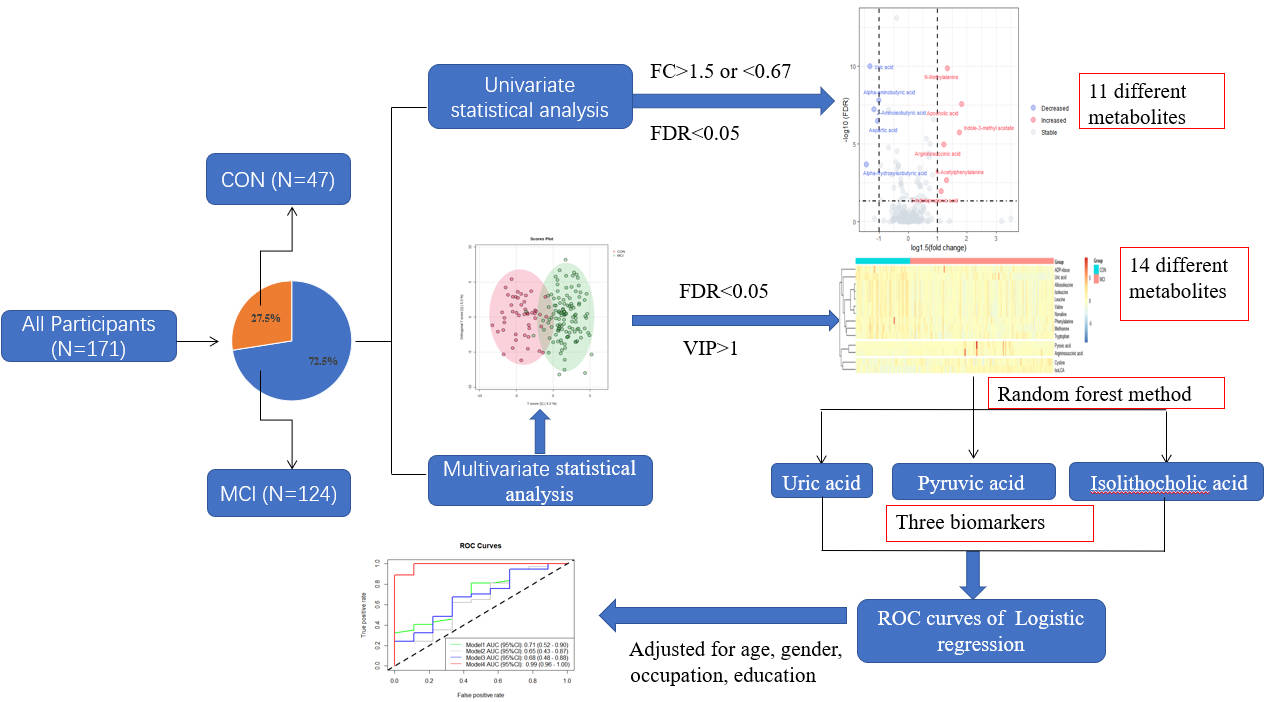


**FIGURE S2**. Gaphical abstract
